# Integration of Sense and Control for Uncertain Systems Based on Delayed Feedback Active Inference

**DOI:** 10.3390/e26110990

**Published:** 2024-11-18

**Authors:** Mingyue Ji, Kunpeng Pan, Xiaoxuan Zhang, Quan Pan, Xiangcheng Dai, Yang Lyu

**Affiliations:** 1 School of Automation, Northwestern Polytechnical University, Xi’an 710129, China; jmy123@mail.nwpu.edu.cn (M.J.); pankunpeng@mail.nwpu.edu.cn (K.P.); xiaoxuanzhang@mail.nwpu.edu.cn (X.Z.); quanpan@nwpu.edu.cn (Q.P.); 2 School of Automation, Nanjing University of Information Science and Technology, Nanjing 210044, China; 202283240029@nuist.edu.cn

**Keywords:** active inference, input delay, predicted state, preference control

## Abstract

Asa result of the time lag in transmission, the data obtained by the sensor is delayed and does not reflect the state at the current moment. The effects of input delay are often overlooked in active inference (AIF), which may lead to significant deviations in state estimation and increased prediction errors, particularly when the system is subjected to a sudden external stimulus. In this paper, a theoretical framework of delayed feedback active inference (DAIF) is proposed to enhance the applicability of AIF to real systems. The probability model of DAIF is defined by incorporating a control distribution into that of AIF. The free energy of DAIF is defined as the sum of the quadratic state, sense, and control prediction error. A predicted state derived from previous states is defined and introduced as the expectation of the prior distribution of the real-time state. A proportional-integral (PI)-like control based on the predicted state is taken to be the expectation of DAIF preference control, whose gain coefficient is inversely proportional to the measurement accuracy variance. To adaptively compensate for external disturbances, a second-order inverse variance accuracy replaces the fixed sensory accuracy of preference control. The simulation results of the trajectory tracking control of a quadrotor unmanned aerial vehicle (UAV) show that DAIF performs better than AIF in state estimation and disturbance resistance.

## 1. Introduction

The human brain can infer and predict hidden states of the world model by minimizing the error between inner and posterior beliefs [1]. Active inference (AIF), proposed by Carl Friston [2], is a comprehensive theory that integrates statistical mechanics and brain science to endow agents with human-like abilities when interacting with the external world [3]. In contrast to prevalent artificial intelligence algorithms such as deep learning and reinforcement learning, AIF does not rely on large amounts of empirical data. Within the framework of AIF, agents perform state inferences against hidden states of the external world model and autonomously take preference actions by minimizing free energy [4].

AIF is a unified theory that integrates information theory and cybernetics [5]. From an information-theoretic perspective, it functions as a Bayesian inference machine that approximates model evidence using variational methods [6]. In practical industrial systems, the presence of model uncertainty and measurement uncertainty cannot be overlooked. The hidden states of uncertain systems can be highly complex, making it challenging to compute all probability distributions of their measurements through Bayesian inference, such as the 2-D distributed pose estimation of multi-agent systems [7]. AIF introduces an approximate distribution to estimate the marginal probabilities of the proposed measurements [8]. From a cybernetic perspective, AIF is a preference control that maximizes expectations [9]. Unlike traditional control algorithms, it does not require the pre-determination of model parameters for AIF preference control. In fact, due to the nonlinear, strong coupling, high-dynamic, and uncertain characteristics of generally uncertain systems, the model parameters can only be approximated as a probability distribution. In utilizing mean-field approximation [10] and Laplace transforms [11], the probability distribution within the Bayesian framework can be approximated as a Gaussian distribution function, transforming the probability model into a linear generative model with noise. The parameters of the generative model are contingent upon the mean of the probability distribution, while estimation accuracy and control hinge on the variance [12].

The mathematical expression of AIF can be seen as a fusion of variational filtering and preference control [13]. Variational filters aim to minimize the variational free energy (VFE) [8], while preference control aims to minimize the expected free energy (EFE) [3]. Although the direct relationship between AIF and traditional control theory has not been proven, it is noteworthy that the free energy, which can be expressed as a form of the quadratic state prediction error and quadratic sense prediction error [14], bears a resemblance to the criterion of the linear quadratic form in linear quadratic regulator (LQR) control [15]. Additionally, the structure of the AIF preference controller shares similarities with a PI controller [16], where the gain coefficients are determined by the inverse variance of the measurement distribution and its derivative. If the system experiences a significant disturbance, it is advisable to employ an adaptive tuning mechanism for setting the gain coefficients of the PI controller to improve its robustness [17,18]. Similarly, the inverse variance precision of the preference control should also be adjusted using an adaptive tuning mechanism rather than fixed constants [16].

**Figure 1:**
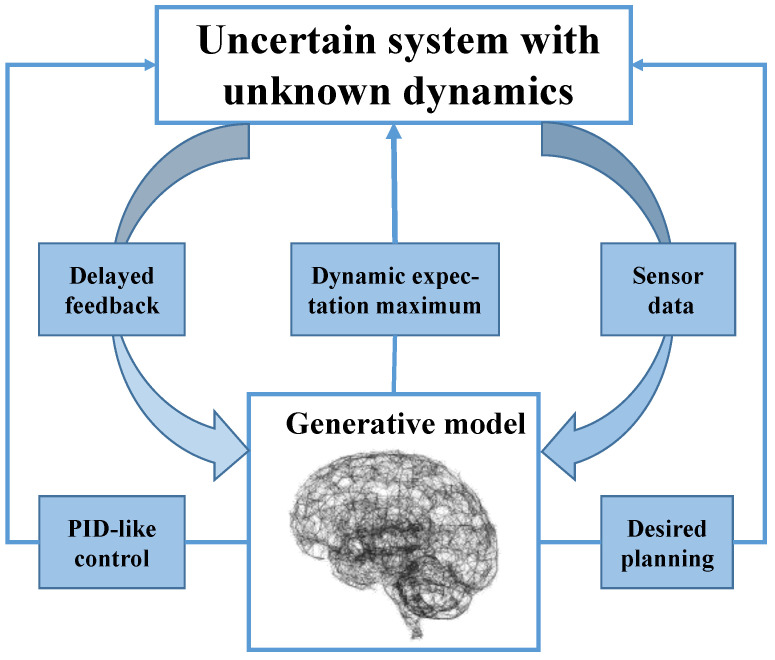
Diagram of the framework of AIF for an uncertain system.

As depicted in Figure 1, AIF, as a general artificial intelligence algorithm, has been widely utilized for the perception, control, and planning of uncertain systems [19]. General systems are inevitably exposed to various uncertain disturbances in complex environments; for instance, a quadrotor UAV may encounter wind and electromagnetic disturbances during operations [20,21,22]. AIF has demonstrated its superiority in agent perception and control. For example, the dynamic expectation maximum (DEM), which outperforms the classical Kalman filter [23] in handling color noise during filtering processes [24], is employed for the state estimation of uncertain systems [25]. Furthermore, AIF preference control [26] surpasses model reference adaptive control in adapting to internal and external parameter perturbations in 7-DOF robotic arm experiments [27]. Agents may autonomously execute preferred actions based on predicted future system states [28]. Maximum expected path planning experiments with mobile robots [29] indicate that hierarchical models within deep AIF exhibit promising potential in more complex environments with partially observable and high-dimensional inputs [30]. Additionally, AIF continues to be optimized as the theory advances, such as through unbiased AIF [31] and multisensory AIF [32].

As a new theory, AIF has some drawbacks. The estimated state is biased toward the target state due to the influence of the target prior, resulting in a degradation of estimation quality [33], such as false positive failures in fault diagnosis and fault tolerance control [16]. The absence of modeling in preference control within AIF may lead to saturation issues. When the approximate model is derived from sensor data of an unknown and uncertain system, input delay is often overlooked in general AIF. The delayed feedback from the world model [34] to the generative model may result in the controller being unable to act on the system promptly due to signal transmission and system reaction delays [35,36]. The delay feedback threatens system security as even a small input delay may cause significant oscillations [37]. Additionally, the state estimation of AIF may reflect the delayed state rather than the current state. The measure-dependent preference control is currently not applicable due to the delayed sensor data obtained in the generative model [38].

Inspired by the literature described above, a novel framework of delayed feedback active inference (DAIF) is proposed for the uncertain system with an input delay. The main contribution of this paper can be summarized as follows:

(1) Compared with AIF, the state estimation of DAIF is proved to not exhibit bias toward the target state. The linear generative model of DAIF for a general uncertain system with an input delay is proposed. The free energy of DAIF is defined as the sum of the quadratic state, sense, and control prediction errors, and the optimized form of state estimation and preference control is given.

(2) Due to this, the data obtained by the sensor are delayed as a result of transmission delay; rather than obtaining real-time data, a predicted state of the current time is defined and obtained by the delayed state and input. They are encoded in the prior state in the generative model of DAIF. The introduction of the predicted state compensates for the AIF’s inability to match real-time data online.

(3) In contrast to AIF, the control distribution is considered in the probabilistic model of DAIF. An epitaxial delayed feedback PI control based on the predicted state is defined and introduced as the expectation of the DAIF preference control. The fixed gain coefficient is replaced by the second-order inverse variance accuracy of the measurement distribution to improve the anti-disturbance performance of the uncertain system.

The main structure of this paper is given as follows: The theoretical framework for AIF is provided in Section 2. The theoretical framework for DAIF is presented in Section 3. Section 4 compares the performance of AIF and DAIF using the simulation of a quadrotor UAV. Finally, the conclusion is summarized in Section 5.

## 2. Preliminaries

AIF is a posterior theory grounded in Bayesian inference (BIF) that utilizes measurements to infer the hidden states of a system, aligning its internal belief with the observed world through preference control. This section presents the theoretical framework and concepts.

### 2.1. Probabilistic Model of AIF

The probability model of AIF is given by

$$p\left(x_{t}|y_{t}\right)=\frac{p\left(y_{t}|x_{t}\right)p\left(x_{t}\right)}{p(y_{t})},$$

where $x_{t}$ and $y_{t}$ are the partially observable hidden states and corresponding measurements at time step *t*. Depending on the research object, $a_{t}$ as the preference control can be torque, force, acceleration, or velocity. $p(x_{t})$, $p(x_{t}|y_{t})$, $p(y_{t})$, and $p(y_{t}|x_{t})$ are the prior, posterior, and marginal probabilities and the likelihood distribution.

**Remark** **1.** *Model and measurement uncertainties are inherent in real systems, leading to the presence of noisy sensory inputs known as partially observable hidden states.*

An approximate posterior distribution $q(z_{t})$ is introduced to infer the posterior distribution $p(x_{t}|y_{t})$ of the hidden states by minimizing the KL divergence as follows:

$$D_{KL}\left(q{\left(z\right)}_{t}∥p(x_{t}|y_{t})\right)=\int q\left(z_{t}\right)\ln\frac{q(z_{t})}{p(x_{t}|y_{t})}dx_{t}.$$

**Remark** **2.** *Calculating the posterior distribution directly is infeasible due to the intricate nature of the marginal probabilities of all hidden states. Therefore, a variational method is proposed for obtaining an approximate posterior distribution. The probability distributions in AIF are commonly assumed to follow Gaussian distributions. The variance reflects the confidence level in the internal belief, with a smaller variance indicating a stronger focus on the expected value.*

### 2.2. Variational Free Energy

**Figure 2:**
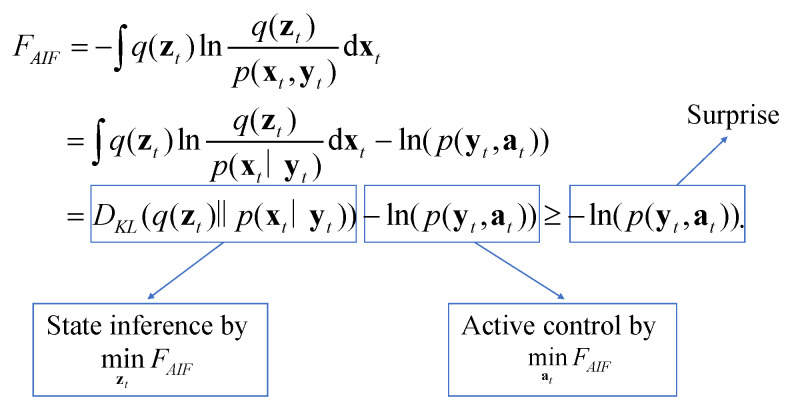
Free energy as the optimization objective for both estimation and control.

Variational free energy (VFE) can also be referred to as free energy and is defined by the KL divergence between the approximate distribution $q(z_{t})$ and joint distribution $p(x_{t},y_{t})$ [39]. As depicted in Figure 2, $F_{AIF}$ can be represented by the KL divergence between $q(z_{t})$ values, and $p(x_{t}|y_{t})$ subtracts the model evidence $\ln\left(p\left(y_{t},a_{t}\right)\right)$:

VFE is an upper bound on the model evidence. Minimizing free energy equates to maximizing model evidence, also referred to as minimization ‘surprise’ [10].

**Remark** **3.** *Minimizing free energy serves as a performance metric, akin to the loss function in deep learning. This process forms a closed loop where state inference induces active control, and active control, in turn, modulates state inference.*

**Figure 3:**
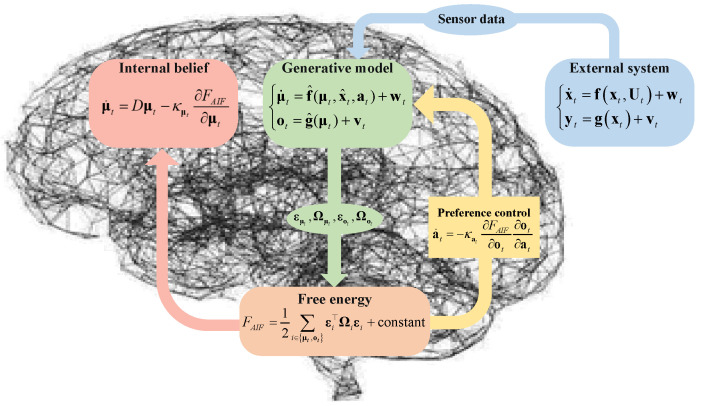
Normal AIF for state estimation and preference control of uncertain system.

The generative model of AIF, as depicted in Figure 3, is constructed based on the sensor data from the external system. The external system, characterized by model uncertainty $w_{t}$ and measurement uncertainty $v_{t}$, is described as follows:

(1) $$\left\{\begin{array}{c} {\dot{x}}_{t}=f\left(x_{t},U_{t}\right)+w_{t},\\ y_{t}=g\left(x_{t}\right)+v_{t}, \end{array}\right.$$

where $f\left(x_{t},U_{t}\right)$ is a linear or nonlinear function dependent on the state $x_{t}$ and control input $U_{t}$, while $g\left(x_{t}\right)$ is also a function of the state. $w_{t}$ and $v_{t}$ are generally assumed to follow Gaussian noise with zero mean as $N\left(0,\Sigma_{\mu_{t}}\right)$ and $N\left(0,\Sigma_{o_{t}}\right)$. $\Sigma_{\mu_{t}}$ and $\Sigma_{o_{t}}$ are the covariance matrices that represent the confidence of inference and control.

As a theory rooted in brain neuroscience, AIF primarily focuses on serving as a generative model. The prior of the hidden state $p(x_{t})$ is represented by a Gaussian distribution with an expectation of $\mu_{t}$. Similarly, the likelihood distribution $p(y_{t}\left|x_{t}\right.)$ is also encoded using a Gaussian distribution with an expectation of $o_{t}$. The generative model for system (1) is expressed by

(2) $$\left\{\begin{array}{c} \dot{\mu_{t}}=\hat{f}\left(\mu_{t},{\hat{x}}_{t},a_{t}\right)+w_{t},\\ o_{t}=\hat{g}\left(\mu_{t}\right)+v_{t}, \end{array}\right.$$

where the generative function $\hat{f}\left(\mu_{t},{\hat{x}}_{t},a_{t}\right)$ is dependent on the state estimation $\mu_{t}$, target state ${\hat{x}}_{t}$, and preference control $a_{t}$, while $\hat{g}\left(\mu_{t}\right)$ represents the mapping function between the state $\mu_{t}$ and sensory input $o_{t}$.

In utilizing the mean-field approximation and Laplace transform [40], the free energy in AIF is characterized as a quadratic linear type of prediction error as follows:

(3) $$F_{AIF}=\frac{1}{2}\sum_{i\in \left\{\mu_{t},o_{t}\right\}}\varepsilon_{i}^{⊤}\Omega_{i}\varepsilon_{i}+constant,$$

where $\varepsilon_{\mu_{t}}={\dot{\mu }}_{t}-\left({\hat{x}}_{t}-\mu_{t}\right)$ and $\varepsilon_{o_{t}}=o_{t}-\mu_{t}$ are the state and sense prediction errors. $\underset{i\in \left\{\mu_{t},o_{t}\right\}}{\Omega_{i}}=\underset{i\in \left\{\mu_{t},o_{t}\right\}}{\Sigma_{i}^{-1}}$ is the inverse variance. ${\varepsilon_{i}}^{T}$ denotes the transpose of the matrix of $\varepsilon_{i}$.

The belief updating and preference control can be described by the optimized format about the gradient descent of free energy as follows [13]:

(4) $$\left\{\begin{array}{c} {\dot{\mu }}_{t}=D\mu_{t}-\kappa_{\mu_{t}}\frac{\partial F_{AIF}}{\partial \mu_{t}}=D\mu_{t}-\kappa_{\mu_{t}}\varepsilon_{\mu_{t}}^{T}\Omega_{\mu_{t}},\\ {\dot{a}}_{t}=-\kappa_{a_{t}}\frac{\partial F_{AIF}}{\partial o_{t}}\frac{\partial o_{t}}{\partial a_{t}}=-\kappa_{a_{t}}\varepsilon_{o_{t}}^{T}\Omega_{o_{t}}\frac{\partial o_{t}}{\partial a_{t}}, \end{array}\right.$$

where $\kappa_{\mu_{t}}$ and $\kappa_{a_{t}}$ are the gradient descent step sizes. *D* is the differential (shift) operator matrix. $\frac{\partial o_{t}}{\partial a_{t}}$ is a mapping that relates the preference control to the sensory input [27].

Agents consistently limit their sensory perception of uncertainty in the external environment to a narrow range of possibilities to counteract the inherent tendency toward disorder [38]. They may update their perception by minimizing free energy and actively provide feedback to the generative model through preference-based behavior driven by prediction error. In AIF, agents continually adjust their expected internal beliefs through ongoing sense–inference–control, aligning more closely with organismic instincts.

**Remark** **4.** *The internal belief in the generative model is also the expected state of the approximate distribution. Minimizing free energy allows for obtaining internal beliefs with the highest confidence. The preference control feedback to the generative model serves as an inverse model that determines the necessary adjustments in actions $a_{t}$ to elicit a corresponding change in sensory observations $o_{t}$. In this manner, actions are generated based on prediction errors, providing a straightforward form of feedback control.*

## 3. The Framework of DAIF

**Figure 4:**
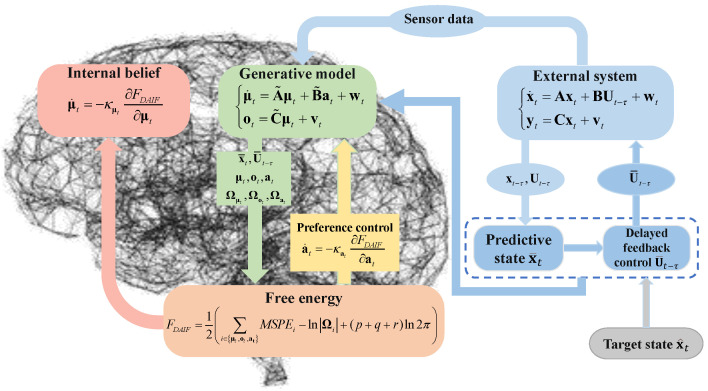
DAIF for state estimation and preference control of uncertain system.

This section presents the framework of delayed feedback active inference (DAIF) for uncertain systems with input delay. In contrast to the normal AIF framework for general systems, DAIF only allows obtaining the prior of the delayed state, not the current state due to input delay. Consequently, a predicted state derived from the delayed state is proposed as the expected value of the state. Additionally, a PI-like delayed feedback control based on the predicted state is introduced as the expected value of preference action in the generative model. The overall framework of DAIF is illustrated in Figure 4.

### 3.1. Probabilistic Model of DAIF

For an uncertain system with hidden state $x_{t}$, measurement $y_{t}$, and delayed control input $U_{t-\tau }$, the probabilistic model is denoted as $p_{t}\left(x_{t},y_{t},U_{t-\tau }\right)$. In contrast to AIF, DAIF considers the control distribution $p\left(U_{t-\tau }|x_{t}\right)$. The probability distribution of the model is factorized accordingly as follows:

(5) $$p_{t}\left(x_{t},y_{t},U_{t-\tau }\right)=p\left(U_{t-\tau }|x_{t}\right)p\left(y_{t}|x_{t}\right)p\left(x_{t}\right).$$

DAIF aims to infer the posterior distribution over the state and control, $p\left(x_{t},U_{t-\tau }|y_{t}\right)$, from the available sensory data. A joint approximate distribution $q(x_{t},U_{t-\tau })$ is introduced for variational inference. The free energy of DAIF can be defined as complexity minus accuracy, as follows:

$$\begin{array}{c} F_{DAIF}=\int q\left(x_{t},U_{t-\tau }\right)\ln\frac{q\left(x_{t},U_{t-\tau }\right)}{p\left(x_{t},y_{t},U_{t-\tau }\right)}dx_{t}\\ \;\;\;=\int q\left(x_{t},U_{t-\tau }\right)\ln\frac{q\left(x_{t},U_{t-\tau }\right)}{p\left(x_{t},U_{t-\tau }|y_{t}\right)}dx_{t}-\ln\left(p\left(y_{t}\right)\right)\\ \;\;\;=D_{KL}\left(q\left(x_{t},U_{t-\tau }\right)∥p(x_{t},U_{t-\tau }|y_{t})\right)-\ln\left(p\left(y_{t}\right)\right). \end{array}$$

where complexity is the KL divergence between $q(x_{t},U_{t-\tau })$ and $p(x_{t},U_{t-\tau }|y_{t})$, while accuracy measures sensory surprisal.

**Remark** **5.** *Input delay is the consequence of signal transmission or delayed response, which is an unavoidable phenomenon. Even a minor delay, if overlooked, can have significant implications for the system and pose a security threat. Therefore, DAIF is more suitable for real uncertain systems than AIF.*

### 3.2. Predicted State

The dynamic model of the uncertain system with an input delay τ can be represented by a nominal system along with model uncertainty $w_{t}$ and measurement uncertainty $v_{t}$, as follows:

(6) $$\left\{\begin{array}{c} {\dot{x}}_{t}={\mathrm{Ax}}_{t}+{\mathrm{BU}}_{t-\tau }+w_{t},\\ y_{t}={\mathrm{Cx}}_{t}+v_{t}, \end{array}\right.$$

where $x_{t}$ and $y_{t}$ are the state and measurement at time *t* with the delayed input $U_{t-\tau }$. $\left(A,C\right)$ is observable, and $\left(A,B\right)$ is controllable.

The internal dynamics of the generative model for system (6) is represented in a similar form as follows:

(7) $$\left\{\begin{array}{c} {\dot{\mu }}_{t}={\tilde{A}\mu }_{t}+{\tilde{B}a}_{t}+w_{t},\\ o_{t}={\tilde{C}\mu }_{t}+v_{t}, \end{array}\right.$$

where the parameters $\left(\tilde{A},\tilde{B},\tilde{C}\right)$ are solely dependent on the nominal system and do not account for model and measurement uncertainty, where $\mu_{t}$, $o_{t}$, and $a_{t}$ represent the expectation of state $x_{t}$, measurement $y_{t}$, and delayed control input $U_{t-\tau }$, respectively. Both of the model parameters of (6) and (7) only pertain to the nominal system, thus satisfying $\tilde{A}=A$, $\tilde{B}=B$, and $\tilde{C}=C$.

**Remark** **6.** *The model parameters of $\left(A,B,C\right)$ that are dependent on the system itself are typically obtained through the process of identifying system parameters. The external or internal uncertainties result in the unknown dynamics of the system (6).*

**Definition** **1.** *With the use of the method of constant variation to solve Equation (6), a predicted state ${\bar{x}}_{t}$ at time t is defined by*

(8) $${\bar{x}}_{t}=e^{A\tau }x_{t-\tau }+\int_{t-\tau }^{t}e^{A\left(t-s\right)}\left[\mathrm{BU}\left(s-\tau \right)+w\left(s\right)\right]ds.$$

*The complex integral $\Gamma_{t}=\int_{t-\tau }^{t}e^{A\left(t-s\right)}\mathrm{BU}\left(s-\tau \right)ds$ can be discretized according to [41]:*

***Step 1:***
*Let T denote the sampling period and τ=NT−η, where N is a positive integer and 0<η<T. When the sampling period is sufficiently small, the control input during each sampling period satisfies*
$$U_{t}=U_{kT},\;kT\leq t<\left(k+1\right)T.$$
  *$\Gamma_{t}$ can be transformed into*
$$\begin{array}{c} \Gamma_{t}=\int_{t-\tau }^{t}e^{A\left(t-s\right)}\mathrm{BU}\left(s-\tau \right)ds\\ \;\;=\int_{-\tau }^{0}e^{-As}\mathrm{BU}\left(s+t-\tau \right)ds\\ \;\;=e^{A(NT-\eta )}\int_{0}^{T-\eta }e^{-As}ds{\mathrm{BU}}_{t-2NT+\eta }+e^{A(N-1)T}\int_{0}^{T}e^{-As}ds{\mathrm{BU}}_{t-(2N-1)T+\eta }\\ \;\;+...+e^{AT}\int_{0}^{T}e^{-As}ds{\mathrm{BU}}_{t-(N+1)T+\eta }. \end{array}$$

***Step 2:***
*Suppose that $\varphi \left(\zeta \right)=\int_{0}^{\zeta }e^{-As}dsB$, $\Gamma_{t}$ can be rewritten as*
$$\begin{array}{c} \Gamma_{t}=e^{A(NT-\eta )}\varphi_{T-\eta }U_{t-2NT+\eta }+e^{A(NT-1)}\varphi_{T}U_{t-2NT+T+\eta }+\ldots +e^{A(T)}\varphi_{T}U_{t-NT-T}. \end{array}$$

***Step 3:***
*Let η=0 and $\Gamma_{t}$ be simplified as*
$$\begin{array}{c} \Gamma_{t}=e^{A(NT)}\varphi_{T}U_{t-2NT}+e^{A(NT-1)}\varphi_{T}U_{t-2NT+T}+\ldots +e^{A(T)}\varphi_{T}U_{t-NT-T}. \end{array}$$
  *Then, the predicted state can be obtained through discrete summation involving the delayed state and control input.*

### 3.3. Preference Control

The target state ${\hat{x}}_{t}$ is encoded in the distribution $p\left(U_{t-\tau }|x_{t}\right)$, which serves as the prior for the DAIF preference control. Since the AIF preference control follows a PI-like control form, the proposed DAIF preference control is expected to follow a similar form. Before designing the control strategy based on this expectation, it is necessary to transform the delay system (6) into a delay-free one through an integral transformation as follows:

(9) $$\psi_{t}=x_{t}+\int_{t-\tau }^{t}e^{-A(s-t+\tau )}\left[\mathrm{BU}\left(s\right)+\omega \left(s\right)\right]ds.$$

and the new state $\psi_{t}$ and original state $x_{t}$ satisfy $\psi_{t}=e^{A\tau }x_{t+\tau }$ [35]. The state equation of the delay-free system is given by

(10) $${\dot{\psi }}_{t}=A\psi_{t}+e^{-A\tau }BU_{t}+e^{-A\tau }\omega_{t}.$$

For system (10), the target state is ${\hat{\psi }}_{t}=e^{A\tau }{\hat{x}}_{t+\tau }$, and the tracking error is $\eta_{t}=\psi_{t}-{\hat{\psi }}_{t}$. In replacing the current time *t* with the delayed time t−τ, the delay feedback control of system (6) is given by

(11) $$\begin{array}{c} {\dot{U}}_{t-\tau }=-\kappa_{a_{t}}\Omega_{o_{t}}\eta_{t-\tau }-\kappa_{{\dot{a}}_{t}}\Omega_{{\dot{o}}_{t}}{\dot{\eta }}_{t-\tau }\\ \begin{array}{c} \;\;=-\kappa_{a_{t}}\Omega_{o_{t}}\left(e^{A\tau }x_{t}-{\hat{\psi }}_{t-\tau }\right)-\kappa_{{\dot{a}}_{t}}\Omega_{{\dot{o}}_{t}}\left(e^{A\tau }{\dot{x}}_{t}-{\dot{\hat{\psi }}}_{t-\tau }\right). \end{array} \end{array}$$

The gain coefficients $\Omega_{o_{t}}$ and $\Omega_{{\dot{o}}_{t}}$ represent the inverse variance of the measurement’s probability density, also referred to as sensory accuracy, which quantifies the deviation between sensory and expectation. They play a crucial role in determining the performance of preference control (11). The proportional term drives the state toward the target state, while the derivative term reduces fluctuations by adjusting the rate of change of the state.

To optimize sense prediction performance, we replace fixed sensory precision with adaptive turning sensory precision. It is promising to substitute sensory accuracy with the logarithmic precision of the inverse variance as follows:

$$\underset{i\in \{o_{t},{\dot{o}}_{t}\}}{{\hat{\Omega }}_{i}}=\ln\underset{i\in \{o_{t},{\dot{o}}_{t}\}}{\Omega_{i}}.$$

We utilize a second-order online adaptive turning scheme based on generalized filtering as described in [16], which is provided by

(12) $$\left\{\begin{array}{c} {\dot{\hat{\Omega }}}_{o_{t}}={{\hat{\Omega }}_{o_{t}}}^{\prime },\\ {{\dot{\hat{\Omega }}}_{o_{t}}}^{\prime }=-\frac{\partial F_{DAIF}}{\partial {\hat{\Omega }}_{o_{t}}}-\kappa_{{\hat{\Omega }}_{o_{t}}}{{\hat{\Omega }}_{o_{t}}}^{\prime }\\ \;=-\frac{1}{2}\left[\exp\left({\hat{\Omega }}_{o_{t}}\right){\varepsilon_{o_{t}}}^{2}-1\right]-\kappa_{{\hat{\Omega }}_{o_{t}}}{{\hat{\Omega }}_{o_{t}}}^{\prime }. \end{array}\right.$$

Due to the input delay, only the delay state $x_{t-\tau }$ in Equation (11) can be perceived at time *t*, but not $x_{t}$. Thus, it is proposed to replace $x_{t}$ with the predicted state ${\bar{x}}_{t}$ in Definition 1, and ${\bar{U}}_{t-\tau }$ as the expectation of the DAIF preference control is given by

(13) $$\begin{array}{c} {\dot{\bar{U}}}_{t-\tau }=-\kappa_{a_{t}}{\hat{\Omega }}_{o_{t}}\left(e^{A\tau }{\bar{x}}_{t}-{\hat{\psi }}_{t-\tau }\right)-\kappa_{{\dot{a}}_{t}}{\hat{\Omega }}_{{\dot{o}}_{t}}\left(e^{A\tau }{\dot{\bar{x}}}_{t}-{\dot{\hat{\psi }}}_{t-\tau }\right). \end{array}$$

All the probability distributions in Equation (5) are assumed to be the multivariate Gaussian distribution as follows:

$$p\left(x_{t}\right)=\sqrt{\frac{\left|\Omega_{\mu_{t}}\right|}{{\left(2\pi \right)}^{p}}}\exp\left(-\frac{1}{2}\varepsilon_{\mu_{t}}^{T}\Omega_{\mu_{t}}\varepsilon_{\mu_{t}}\right),$$

$$p\left(y_{t}|x_{t}\right)=\sqrt{\frac{\left|\Omega_{o_{t}}\right|}{{\left(2\pi \right)}^{q}}}\exp\left(-\frac{1}{2}\varepsilon_{o_{t}}^{T}\Omega_{o_{t}}\varepsilon_{o_{t}}\right),$$

$$p\left(U_{t-\tau }|x_{t}\right)=\sqrt{\frac{\left|\Omega_{a_{t}}\right|}{{\left(2\pi \right)}^{r}}}\exp\left(-\frac{1}{2}\varepsilon_{a_{t}}^{T}\Omega_{a_{t}}\varepsilon_{a_{t}}\right),$$

where $\varepsilon_{\mu_{t}}=\mu_{t}-{\bar{x}}_{t}$, $\varepsilon_{o_{t}}=o_{t}-\tilde{C}\mu_{t}$, and $\varepsilon_{a_{t}}=a_{t}-{\bar{U}}_{t-\tau }$ are the state prediction error, sense prediction error, and control prediction error. The free energy function of DAIF is given by

(14) $$\begin{array}{c} F_{DAIF}=\frac{1}{2}\sum_{i\in \left\{\mu_{t},o_{t},a_{t}\right\}}\left(\varepsilon_{i}^{⊤}\Omega_{i}\varepsilon_{i}-\ln\left|\Omega_{i}\right|\right)+\frac{1}{2}\left(p+q+r\right)\ln2\pi . \end{array}$$

where *p*, *q*, and *r* are, respectively, the dimensions of vectors $\mu_{t}$, $o_{t}$, and $a_{t}$.

The state estimation and preference control of DAIF can be achieved using gradient descent on $F_{DAIF}$ like AIF as follows:

(15) $$\left\{\begin{array}{c} {\dot{\mu }}_{t}=-\kappa_{\mu_{t}}\frac{\partial F_{DAIF}}{\partial \mu_{t}}=-\kappa_{\mu_{t}}\Omega_{\mu_{t}}\left(\mu_{t}-{\bar{x}}_{t}\right),\\ {\dot{a}}_{t}=-\kappa_{a_{t}}\frac{\partial F_{DAIF}}{\partial a_{t}}=-\kappa_{a_{t}}\Omega_{a_{t}}\left(a_{t}-{\bar{U}}_{t-\tau }\right). \end{array}\right.$$

**Remark** **7.** *The free energy of DAIF encodes the expected state, measurement, and control input, unlike AIF. Therefore, minimizing the free energy can be interpreted as reducing the prediction error of the state and sense using minimal energy, which is formally similar to the linear quadratic optimal criterion of LQR control. The added control prediction error in Equation (14) biases the preference for control toward expectation when minimizing free energy.*

### 3.4. Convergence Analysis of AIF and DAIF

**Figure 5:**
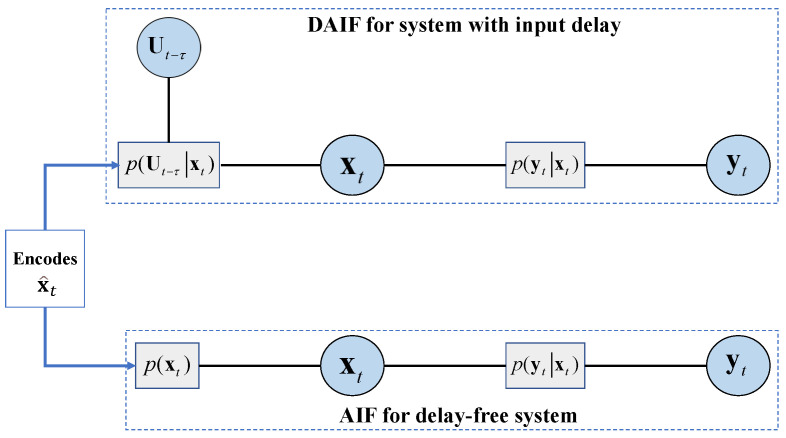
Factor graphs of DAIF (**above**) and AIF (**below**).

As depicted in Figure 5, we provide a visual explanation of the generative probabilistic model using factor graphs. In AIF, the target state ${\hat{x}}_{t}$ is encoded in the prior $p\left(x_{t}\right)$, whereas it is encoded in the conditional probability $p\left(U_{t-\tau }\left|x_{t}\right.\right)$ in DAIF. The preference control is not explicitly modeled as a random variable in the generative model of the AIF [2], and it is directly related to the sensory inputs [38]. In contrast to AIF, DAIF’s probabilistic model takes into account the control distribution $p\left(U_{t-\tau }|x_{t}\right)$.

**Theorem** **1.** *The stable value of the estimated state in AIF is contingent upon the target signal. In contrast, the convergence value in DAIF is not biased toward the target signal but rather depends on the predicted state.*

**Proof** **of Theorem 1** The steady-state expectation $\mu^{*}$ in AIF and DAIF can be obtained by solving the derivative of free energy function (3) and (14) with respect to the estimated state as follows:

(16) $$\frac{\partial F_{AIF}}{\partial \mu_{t}}=0\Rightarrow \mu_{AIF}^{*}=\frac{\Omega_{\mu_{t}}o_{t}+\Omega_{o_{t}}\left({\hat{x}}_{t}-\mu_{t}\right)}{\Omega_{\mu_{t}}+\Omega_{o_{t}}},$$

(17) $$\frac{\partial F_{DAIF}}{\partial \mu_{t}}=0\Rightarrow \mu_{DAIF}^{*}=\frac{\Omega_{\mu_{t}}o_{t}+\Omega_{o_{t}}{\bar{x}}_{t}}{\Omega_{\mu_{t}}+\Omega_{o_{t}}}.$$

The convergence value of the AIF’s steady-state expectation is influenced by the target state ${\hat{x}}_{t}$, as indicated by Formulas (16) and (17). Consequently, when the system experiences a sudden disturbance or sensor failure, it may not provide timely feedback to the generative model due to inevitable input delay. This could result in a significant deviation from the desired optimal state at the present moment. However, the convergence value of the AIF’s steady-state expectation is associated with the predicted state ${\bar{x}}_{t}$ and remains unbiased toward the target signal, thereby compensating for the limitation of AIF. □

## 4. Results

### 4.1. Trajectory Tracking Control of a Quadrotor UAV

We employed a simulation example of a quadrotor UAV to contrast the performance of AIF and DAIF in state estimation and preference control for an uncertain system with input delay. The UAV system’s parameters and corresponding values in this paper are detailed in Table 1. The dynamic model of the quadrotor UAV can be obtained as in [42]:

(18) $$\left\{\begin{array}{c} \ddot{x}=-\frac{K_{1}}{m}\dot{x}+\frac{U_{1}}{m}\left(\cos\psi \sin\theta \cos\phi +\sin\psi \sin\phi \right),\\ \ddot{y}=-\frac{K_{2}}{m}\dot{y}+\frac{U_{1}}{m}\left(\sin\psi \sin\theta \cos\phi -\cos\psi \sin\phi \right),\\ \ddot{z}=-\frac{K_{3}}{m}\dot{z}+\frac{U_{1}}{m}\cos\theta \cos\phi -g,\\ \overset{..}{\phi }=-\frac{K_{4}l}{I_{z}}\dot{\phi }+\frac{l}{I_{x}}U_{2}+\dot{\theta }\dot{\psi }\frac{I_{y}-I_{z}}{I_{x}}+\frac{J_{r}}{I_{x}}\dot{\theta }\xi_{r},\\ \ddot{\theta }=-\frac{K_{5}l}{I_{z}}\dot{\theta }+\frac{l}{I_{y}}U_{3}+\dot{\phi }\dot{\psi }\frac{I_{z}-I_{x}}{I_{y}}-\frac{J_{r}}{I_{y}}\dot{\phi }\xi_{r},\\ \ddot{\psi }=-\frac{K_{6}l}{I_{z}}\dot{\psi }+\frac{U_{4}}{I_{z}}+\dot{\phi }\dot{\theta }\frac{I_{x}-I_{y}}{I_{z}}. \end{array}\right.$$

The three Euler angles ϕ,θ, and ψ represent the roll, the pitch, and the yaw. They satisfy ϕ∈(−π/2,π/2), θ∈(−π/2,π/2), and ψ∈(−π,π).

**Figure 6:**
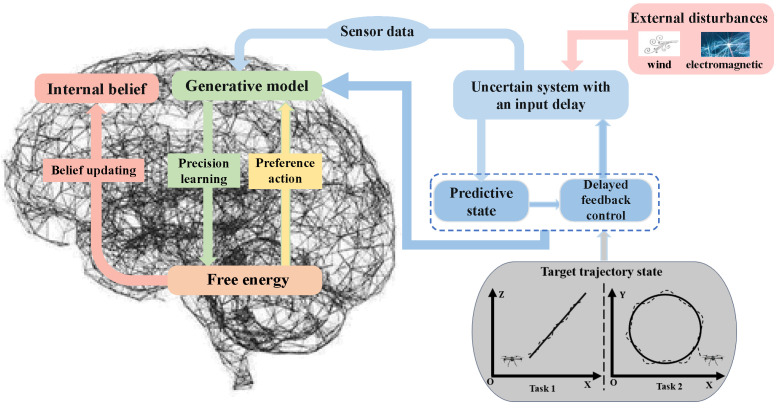
Diagram of trajectory tracking control of the quadrotor UAV based on DAIF.

Figure 6 illustrates the trajectory tracking control diagram of the quadrotor utilizing DAIF. During task execution, the UAV is inevitably subjected to external disturbances such as wind or electromagnetic interference, resulting in model and measurement uncertainty. Abrupt disturbances can lead to pulse-like control inputs or even system failure. Minimizing energy consumption during sudden disturbances poses a significant challenge for the system. In the classical AIF framework, feedback delays may impact estimation and control performance due to the neglect of input delay. Through experiments on the linear and circular target tracking of UAVs, we conducted a comprehensive comparison between AIF and DAIF, while also analyzing the effects of inverse variance accuracy and input delay within the DAIF framework.

#### 4.1.1. Linear Target Trajectory Tracking

It is assumed that the linear target trajectory in the X-O-Z plane is represented by the equation x−z+1=0, and the quadrotor UAV begins from the initial position $\left(-1,-1\right)$. The linear motion of the quadrotor UAV is primarily dependent on $U_{1}$ and $U_{3}$. Assuming that the roll angle ϕ and yaw angle ψ of system (18) are 0 radians, we obtain a linear motion system of UAV as follows

(19) $$\left\{\begin{array}{c} \ddot{x}=-\frac{K_{1}}{m}\dot{x}+\frac{U_{1}}{m}\sin\theta ,\\ \ddot{z}=-\frac{K_{3}}{m}\dot{z}+\frac{U_{1}}{m}\cos\theta -g,\\ \ddot{\theta }=-\frac{K_{5}l}{I_{z}}\dot{\theta }+\frac{l}{I_{y}}U_{3}, \end{array}\right.$$

Let $\lambda_{t}={\left[\begin{array}{cccccc} x & \dot{x} & z & \dot{z} & \theta & \dot{\theta } \end{array}\right]}^{T}$ be the state and $\rho_{t}$ be the measurement vector. System (19) can be rewritten in matrix form with input delay τ, model uncertainty $w_{t}$, and measurement uncertainty $v_{t}$ as follows

(20) $$\left\{\begin{array}{c} {\dot{\lambda }}_{t}={A_{1}\lambda }_{t}+B_{1}U_{t-\tau }+w_{t},\\ \rho_{t}=C_{1}\lambda_{t}+v_{t}, \end{array}\right.$$

where $A_{1}=\left[\begin{array}{cccccc} 0 & 1 & 0 & 0 & 0 & 0\\ 0 & -\frac{K_{1}}{m} & 0 & 0 & 0 & 0\\ 0 & 0 & 0 & 1 & 0 & 0\\ 0 & 0 & 0 & -\frac{K_{3}}{m} & 0 & 0\\ 0 & 0 & 0 & 0 & 0 & 1\\ 0 & 0 & 0 & 0 & 0 & -\frac{K_{5}l}{I_{z}} \end{array}\right]$, $B_{1}={\left[\begin{array}{cccccc} 0 & \frac{\sin\theta }{m} & 0 & \frac{\cos\theta }{m} & 0 & 0\\ 0 & 0 & 0 & 0 & 0 & \frac{l}{I_{y}} \end{array}\right]}^{T}$,

$U_{t-\tau }=\left[\begin{array}{c} U_{1}\left(t-\tau \right)\\ U_{3}\left(t-\tau \right) \end{array}\right]$, and $C_{1}=I_{6\times 6}$. $I_{6\times 6}$ is a sixth-order identity matrix.

The generative model of system (20) is supposed to be

(21) $$\left\{\begin{array}{c} {\dot{\mu }}_{t}=A_{1}\mu_{t}+B_{1}a_{t}+w_{t},\\ o_{t}=C_{1}\mu_{t}+v_{t}, \end{array}\right.$$

where $\mu_{t}={\left[\begin{array}{cccccc} \mu_{1} & {\dot{\mu }}_{1} & \mu_{2} & {\dot{\mu }}_{2} & \mu_{3} & {\dot{\mu }}_{3} \end{array}\right]}^{T}$ and $o_{t}={\left[\begin{array}{cccccc} o_{1} & {\dot{o}}_{1} & o_{2} & {\dot{o}}_{2} & o_{3} & {\dot{o}}_{3} \end{array}\right]}^{T}$ are the expectations of the state and measurement. $a_{t}={\left[\begin{array}{cc} a_{1} & a_{2} \end{array}\right]}^{T}$ is the preference control. The inverse variance precision, prediction error, and free energy are denoted by $\Omega_{i}=\Omega_{i}\cdot I_{6\times 6}$, $\varepsilon_{i}$, and $F_{1}$, where $i\in \left\{\mu_{t},o_{t},a_{t}\right\}$.

**Figure 7:**
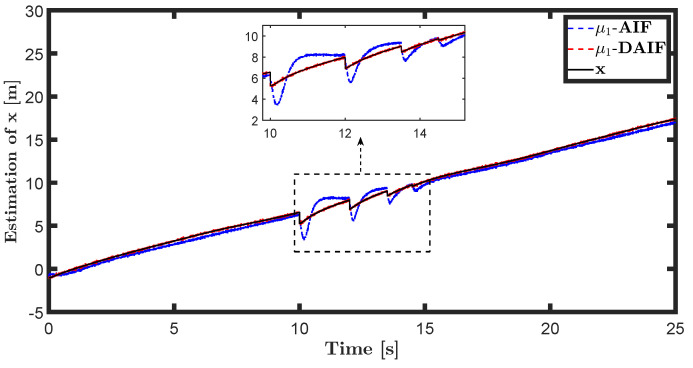
State estimation of *x* in system (19).

**Figure 8:**
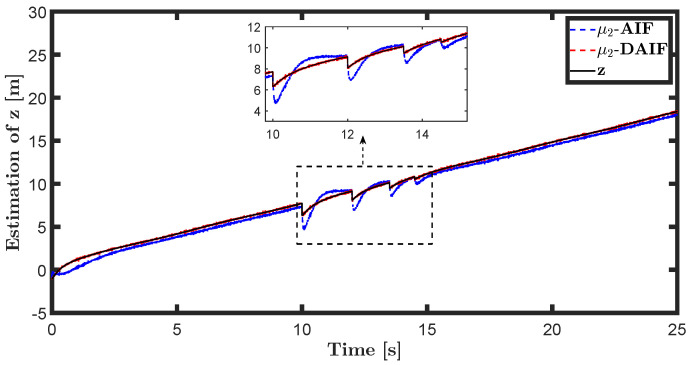
State estimation of *z* in system (19).

**Figure 9:**
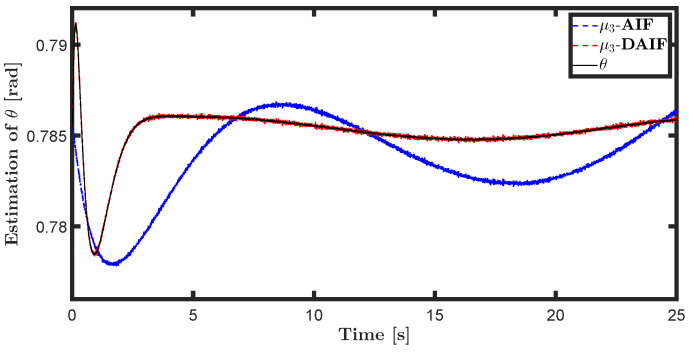
State estimation of θ in system (19).

**Figure 10:**
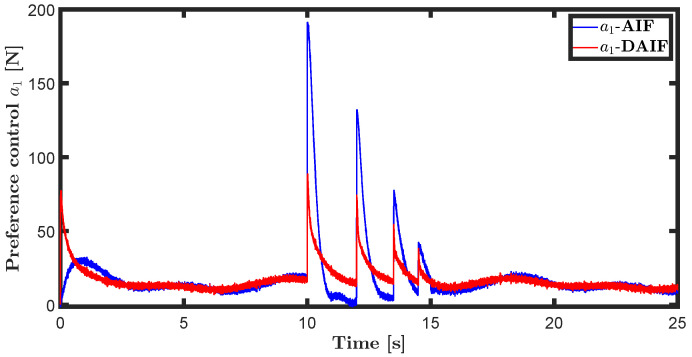
Preference control of the generative model (21).

**Figure 11:**
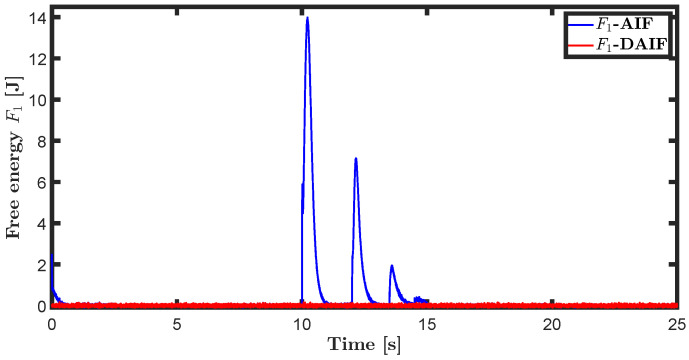
Free energy of the generative model (21).

**Figure 12:**
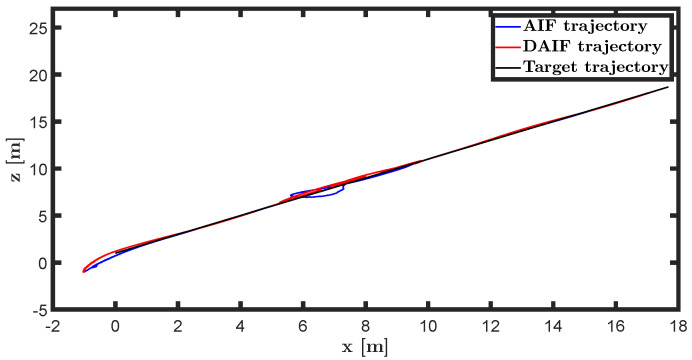
Linear motion trajectory of UAV in X-O-Z plane.

The trajectory tracking target vector of system (20) is set to be ${\tilde{\lambda }}_{t}={\left[\tilde{x},\tilde{z},\tilde{\theta }\right]}^{T}={\left[\frac{\sqrt{2}}{2}t,\frac{\sqrt{2}}{2}t+1,\frac{\pi }{4}\right]}^{T}$. The learning parameters satisfy $\kappa_{\mu }=100$, $\kappa_{a}=200$ and $\kappa_{{\hat{\Omega }}_{o_{t}}}=8$. All the probability distributions are assumed to be the standard Gaussian distribution. The inverse variance precision is $\Omega_{i}=e^{0}$. The total simulation time is 25 s, and the step size is set to 0.001 s. It is assumed that the total thrust $U_{1}$ is subject to a stochastic excitation that is described by $\delta_{t}\in \left[40,80\right]$ during the time interval of 10 s to 15 s, which may be sudden disturbances from wind or electromagnetic sources. The simulation results are shown in Figure 7, Figure 8, Figure 9, Figure 10, Figure 11 and Figure 12.

#### 4.1.2. Circular Target Trajectory Tracking

We assumed that the UAV follows a circular path with a radius of 10 m in the X-Y plane, centered at $\left(0,0\right)$, and represented by the function $x^{2}+y^{2}=100$. The initial position is $\left(12,0\right)$. The yaw angle of the UAV increases uniformly at a rate of 0.28 rad/s. It is assumed that the roll angle ϕ of system (18) is 0 radians, and the curved motion system is obtained by

(22) $$\left\{\begin{array}{c} \ddot{x}=-\frac{K_{1}}{m}\dot{x}+\frac{U_{1}}{m}\sin\theta \cos\psi ,\\ \ddot{y}=-\frac{K_{2}}{m}\dot{y}+\frac{U_{1}}{m}\sin\theta \sin\psi ,\\ \ddot{\psi }=-\frac{K_{6}l}{I_{z}}\dot{\psi }+\frac{U_{4}}{I_{z}}. \end{array}\right.$$

Let ${\lambda^{\prime }}_{t}={\left[\begin{array}{cccccc} x & \dot{x} & y & \dot{y} & \psi & \dot{\psi } \end{array}\right]}^{T}$ and ${\rho^{\prime }}_{t}$ be the state and measurement vector. Equation (22) can be rewritten as the matrix form with input delay τ, model uncertainty $w_{t}^{\prime }$, and measurement uncertainty $v_{t}^{\prime }$ as follows

(23) $$\left\{\begin{array}{c} {{\dot{\lambda }}^{\prime }}_{t}=A_{2}{\lambda^{\prime }}_{t}+B_{2}{U^{\prime }}_{t-\tau }+{w^{\prime }}_{t},\\ {\rho^{\prime }}_{t}=C_{2}{\lambda^{\prime }}_{t}+{v^{\prime }}_{t}, \end{array}\right.$$

where $A_{2}=\left[\begin{array}{cccccc} 0 & 1 & 0 & 0 & 0 & 0\\ 0 & -\frac{K_{1}}{m} & 0 & 0 & 0 & 0\\ 0 & 0 & 0 & 1 & 0 & 0\\ 0 & 0 & 0 & -\frac{K_{2}}{m} & 0 & 0\\ 0 & 0 & 0 & 0 & 0 & 1\\ 0 & 0 & 0 & 0 & 0 & -\frac{K_{6}l}{I_{z}} \end{array}\right]$, $B_{2}={\left[\begin{array}{cccccc} 0 & \frac{\sin\theta \cos\psi }{m} & 0 & \frac{\cos\theta \sin\psi }{m} & 0 & 0\\ 0 & 0 & 0 & 0 & 0 & \frac{1}{I_{z}} \end{array}\right]}^{T}$,

${U^{\prime }}_{t-\tau }=\left[\begin{array}{c} U_{1}\left(t-\tau \right)\\ U_{4}\left(t-\tau \right) \end{array}\right]$, and $C_{2}=I_{6}$.

The generative model of system (23) is supposed to be

(24) $$\left\{\begin{array}{c} {{\dot{\mu }}^{\prime }}_{t}=A_{2}{\mu^{\prime }}_{t}+B_{2}{a^{\prime }}_{t}+{w^{\prime }}_{t},\\ {o^{\prime }}_{t}=C_{2}{\mu^{\prime }}_{t}+{v^{\prime }}_{t}, \end{array}\right.$$

where ${\mu^{\prime }}_{t}={\left[\begin{array}{cccccc} \mu_{4} & {\dot{\mu }}_{4} & \mu_{5} & {\dot{\mu }}_{5} & \mu_{6} & {\dot{\mu }}_{6} \end{array}\right]}^{T}$ and ${o^{\prime }}_{t}={\left[\begin{array}{cccccc} o_{4} & {\dot{o}}_{4} & o_{5} & {\dot{o}}_{5} & o_{6} & {\dot{o}}_{6} \end{array}\right]}^{T}$ are the expectation of the state and measurement. The preference control is ${a^{\prime }}_{t}={\left[\begin{array}{cc} a_{3} & a_{4} \end{array}\right]}^{T}$. The inverse variance precision, prediction error, and free energy are denoted by $\Omega_{i}=\Omega_{i}\cdot I_{6\times 6}$, $\varepsilon_{i}$, and $F_{2}$, where $i\in \left\{{\mu^{\prime }}_{t},{o^{\prime }}_{t},{a^{\prime }}_{t}\right\}$.

**Figure 13:**
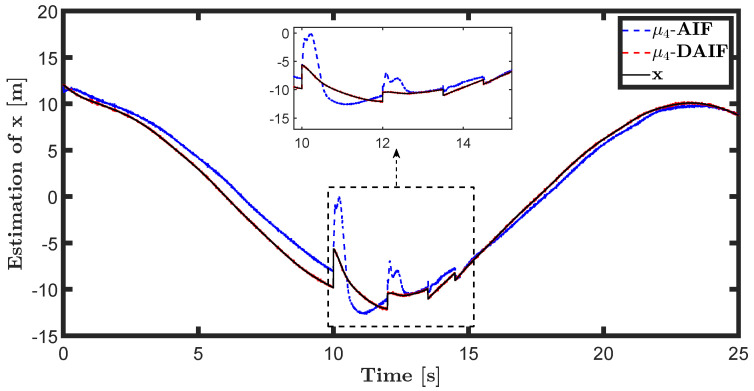
State estimation of *x* in system (22).

**Figure 14:**
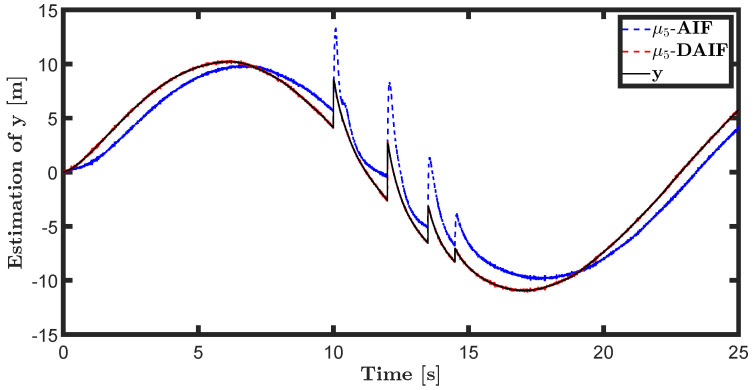
State estimation of *y* in system (22).

**Figure 15:**
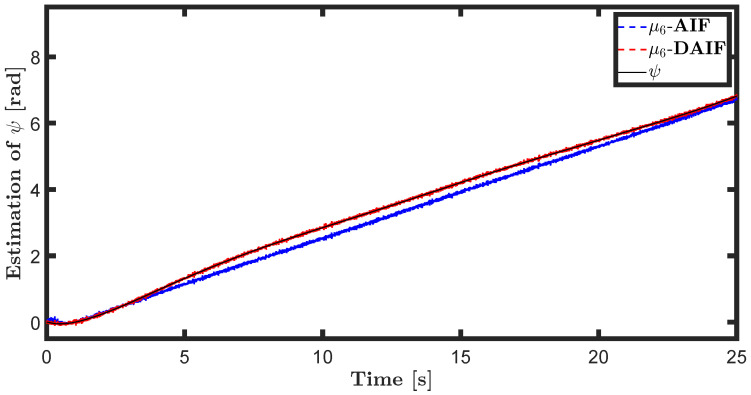
State estimation of ψ in system (22).

**Figure 16:**
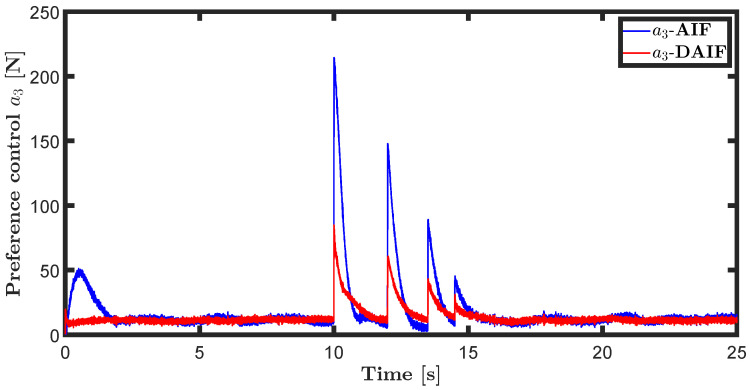
Preference control of the generative model (24).

**Figure 17:**
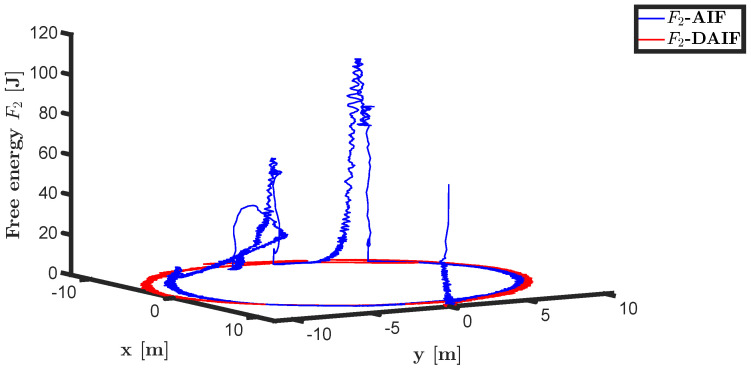
Free energy of the generative model (24).

**Figure 18:**
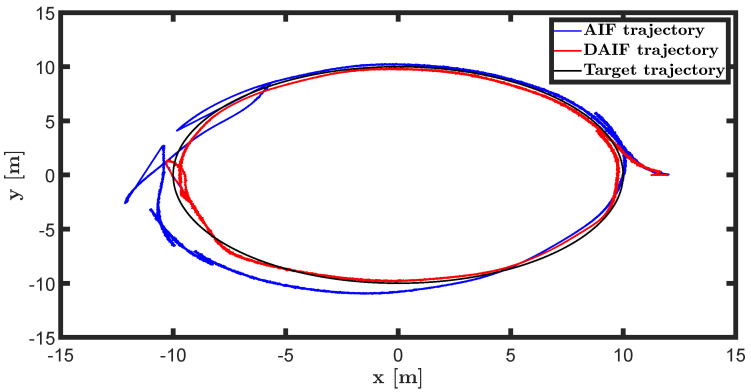
Circular motion trajectory of UAV in X-O-Y plane.

The trajectory tracking target vector of system (23) is set to be ${{\tilde{\lambda }}^{\prime }}_{t}={\left[\tilde{x},\tilde{y},\tilde{\psi }\right]}^{T}={\left[10\cos(0.28t),10\sin(0.28t),0.28t\right]}^{T}$. It is also assumed that the total thrust $U_{1}$ is subject to a stochastic excitation that is represented by $\delta_{t}\in \left[40,80\right]$ during the time interval of 10 s to 15 s. The other parameters are set to be the same as the linear motion of the quadrotor UAV in the X-O-Z plane. The simulation results are shown in Figure 13, Figure 14, Figure 15, Figure 16, Figure 17 and Figure 18.

#### 4.1.3. Discussion

The state estimations for systems (19) and system (22) in the generative model of AIF and DAIF when τ=0.1 s are depicted in Figure 7, Figure 8 and Figure 9 and Figure 13, Figure 14 and Figure 15. Despite the pitch input $U_{3}$ and yawing input $U_{4}$ not being intended to experience sudden disturbances, the estimation for θ and ψ in AIF lags and deviates from the real states compared to DAIF. The estimations for the position coordinates of the quadrotor UAV fluctuate when subjected to a sudden disturbance at 10 s. It is evident that the state estimations in AIF exhibit more fluctuations and take longer to recover than those in DAIF. It is important to note that the generative model is derived from data in the delayed state from the sensor, and measure-dependent preference control essentially involves delayed feedback in AIF, resulting in long lag errors that hinder adaptation to large attacks or disturbances. As shown in Figure 13 and Figure 14, the effects of lag error appear greater in estimating nonlinear targets. Utilizing real-time predicted state and delay-feedback PI control as expectations for state estimation and preference control leads to better anti-disturbance performance in DAIF.

Time history diagrams for preference controls $a_{1}$ and $a_{3}$ are depicted in Figure 10 and Figure 16. When sudden perturbations occur, both $a_{1}$ and $a_{3}$ undergo mutations. These preference-controlled mutations can be seen as the agent’s spontaneous response to disturbances. In neglecting the impact of input delay, AIF requires more energy consumption due to accumulated lag error, resulting in greater mutation of preference control compared to DAIF. As depicted in Figure 11 and Figure 17, DAIF effectively mitigates this disparity, resulting in consistently minimal and stable free energy even in the presence of sudden perturbations.

The trajectories of the quadrotor UAV in linear and circular motion are depicted in Figure 12 and Figure 18, respectively. Utilizing second-order adaptive sensory accuracy as the coefficient of preference control allows the UAV to exhibit anti-disturbance performance in AIF and DAIF. Due to delayed measurement data not aligning with the real-time state, AIF is unable to immediately compensate for sudden perturbations, resulting in a relaxation process for the quadrotor UAV. In comparison, the quadcopter UAV demonstrates superior anti-disturbance performance in DAIF as the predicted state closely mirrors the real-time state.

### 4.2. Precision Learning and Delay Size Analysis

**Figure 19:**
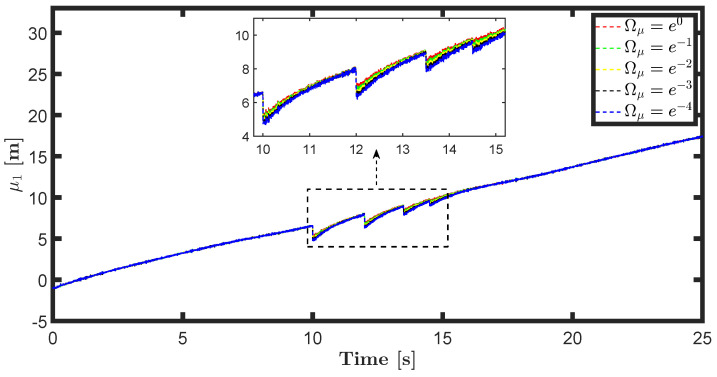
State estimation $\mu_{1}$ for different prediction accuracy $\Omega_{\mu }$.

**Figure 20:**
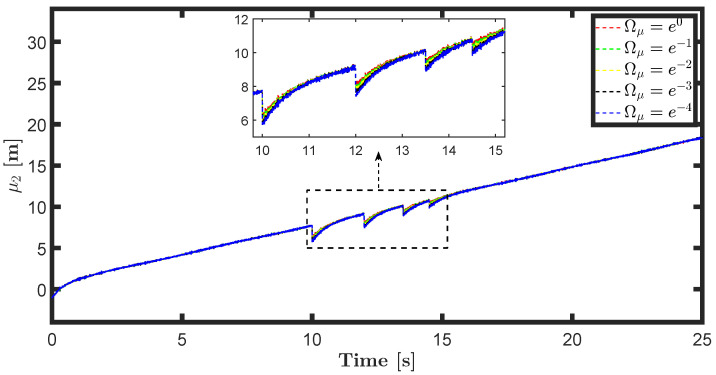
State estimation $\mu_{2}$ for different prediction accuracy $\Omega_{\mu }$.

**Figure 21:**
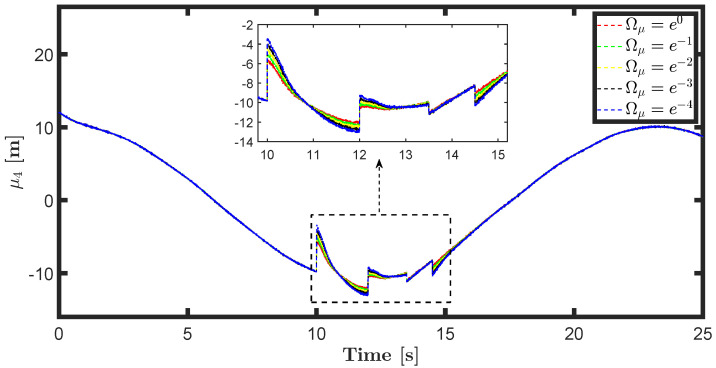
State estimation $\mu_{4}$ for different prediction accuracy $\Omega_{\mu^{\prime }}$.

**Figure 22:**
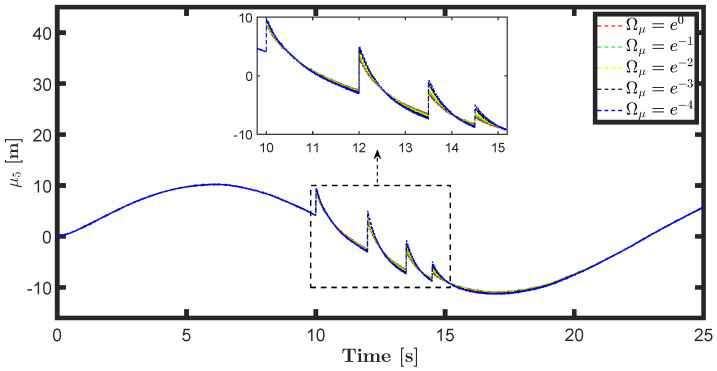
State estimation $\mu_{5}$ for different prediction accuracy $\Omega_{\mu^{\prime }}$.

The state estimations for different prediction precision values in DAIF are illustrated in Figure 19, Figure 20, Figure 21 and Figure 22. It is evident that as $\Omega_{\mu }$ and $\Omega_{\mu^{\prime }}$ increase from $e^{-4}$ to $e^{0}$, the mutation of the state estimates decreases and the system state can return to normal more rapidly. Prediction accuracy essentially represents the inverse variance of the approximate distribution. Greater prediction accuracy can reduce estimation uncertainty and bring the estimated state closer to the predicted state.

**Figure 23:**
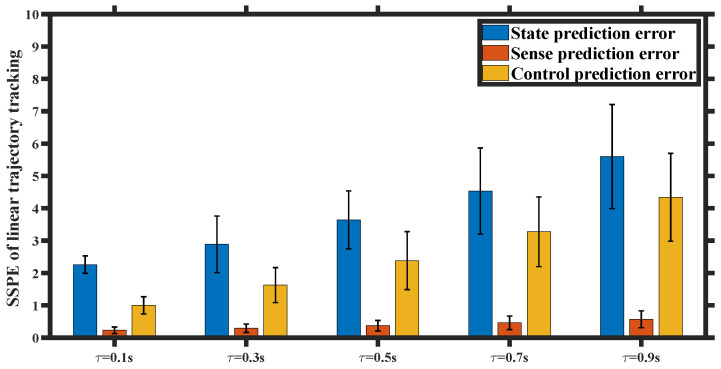
SSPE of linear trajectory tracking for different input delay τ.

**Figure 24:**
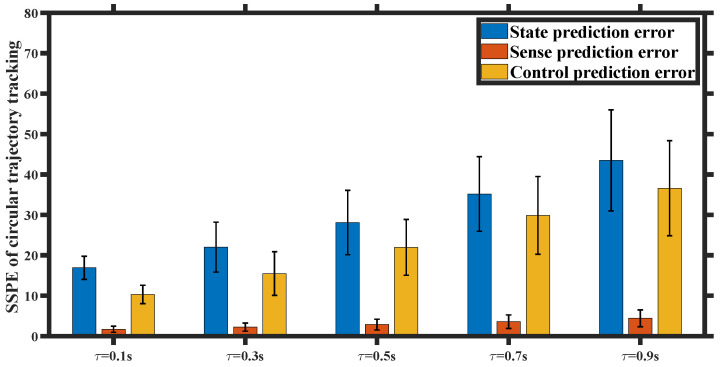
SSPE of circular trajectory tracking for different input delay τ.

We set the state prediction accuracy to be $\Omega_{\mu }=\Omega_{\mu^{\prime }}=1$ and gradually increased the input delay from 0.1 s to 0.9 s. We conducted 5 sets of UAV trajectory tracking experiments with varying input delays, each set consisting of 20 trials. We quantify the predictive performance using the sum of the squared prediction error (SSPE) in state, sense, and control. As depicted in Figure 23 and Figure 24, significant state and control prediction errors were observed as a result of the sudden disturbance-induced mutation in the predicted state and delayed feedback preference control. However, adaptive prediction accuracy led to a minimal perception prediction error. The error bar plot of the SSPE indicates that both the state prediction error and control prediction error increase with higher input delay, highlighting the significance of considering input delay in our analysis.

## 5. Conclusions

This paper introduces a novel DAIF algorithm for uncertain systems with input delay. This algorithm incorporates a predicted state as the current state expectation, designs a delayed feedback PI control as the preferred control expectation, and implements an adaptive tuning mechanism with a gain coefficient. Both AIF and DAIF are utilized for the trajectory tracking of a quadrotor UAV. Simulation results demonstrated that the estimation accuracy of DAIF based on predicted states is higher than that of AIF, and the DAIF preference control performs better than AIF when the uncertain system with an input delay is subjected to sudden disturbance. The existence of input delay will increase the prediction error, so it cannot be ignored.

## Figures and Tables

**Table 1:** The descriptions and values of the UAV system’s parameters.

Symbol	Description	Value
*m*	the total mass	2 kg
*g*	the acceleration of gravity	9.8 $m/s^{2}$
*l*	the centrifugal pitch of the UAV	0.2 m
$K_{i}$	the drag coefficients	0.01 N/m
$I_{x},I_{y},I_{z}$	the inertias of the quadrotor UAV	1.5 $Nm^{2}$/rad
$J_{r}$	the inertia of the propeller	1 $Ns^{2}$/rad
$\xi_{r}$	the angular speed of the propeller	-
${\left[x,y,z\right]}^{T}$	the position of the quadrotor UAV	-
${\left[\phi ,\theta ,\psi \right]}^{T}$	the three Euler angles	-
$U_{1}$	the total thrust	-
$U_{2}$	the roll input	-
$U_{3}$	the pitch input	-
$U_{4}$	the yawing input	-

## Data Availability

The data presented in this study are available on request from the corresponding author.
